# Optimization of Compression Molding Parameters and Lifecycle Carbon Impact Assessment of Bamboo Fiber-Reinforced Polypropylene Composites

**DOI:** 10.3390/polym16233435

**Published:** 2024-12-06

**Authors:** Wei Li, Tao Feng, Tongyuan Lu, Feng Zhao, Jialong Zhao, Wei Guo, Lin Hua

**Affiliations:** 1 Hubei Key Laboratory of Advanced Technology for Automotive Components, Wuhan University of Technology, Wuhan 430070, China; 2 Hubei Collaborative Innovation Center for Automotive Components Technology, Wuhan University of Technology, Wuhan 430070, China; 3 SAIC-GM-Wuling Automobile Co., Ltd., Liuzhou 545007, China; 4 Hubei Research Center for New Energy & Intelligent Connected Vehicle, Wuhan University of Technology, Wuhan 430070, China; 5 Institute of Advanced Materials and Manufacturing Technology, Wuhan University of Technology, Wuhan 430070, China

**Keywords:** bamboo fiber-reinforced composites, compression molding process, polypropylene, life cycle assessment, low-carbon automotive materials

## Abstract

Driven by global carbon neutrality goals, bamboo fiber-reinforced PP composites have shown significant potential for automotive applications due to their renewability, low carbon emissions, and superior mechanical properties. However, the environmental complexities associated with compression molding process parameters, which impact material properties and carbon emissions, pose challenges for large-scale adoption. This study systematically optimized the compression molding process of bamboo fiber-reinforced PP composites through a three-factor, five-level experimental design, focusing on preheating temperature, preheating time, and holding time. Additionally, an innovative life cycle assessment (LCA) was conducted to evaluate the environmental impact. The results indicated that at a preheating temperature of 220 °C, preheating time of 210–240 s, and holding time of 40–50 s, the material achieved a tensile strength of 35 MPa and a flexural strength of 45 MPa, with a 15% reduction in water absorption. The LCA further highlighted energy consumption, the compression molding process, and material composition as the primary contributors to carbon emissions and environmental impacts, identifying key areas for future optimization. This study provides an optimized framework for compression molding bamboo fiber-reinforced PP composites and establishes a theoretical foundation for their low-carbon application in the automotive industry. Future work will explore the optimization of bamboo fiber content and process parameters to further enhance material performance and reduce environmental impact.

## 1. Introduction

With increasing global environmental awareness and stricter regulations, the widespread application of traditional petrochemical-based materials faces mounting challenges [1,2,3]. These materials not only contribute substantial carbon emissions during production and use but also pose long-term environmental risks due to their poor degradability [4]. As a result, the pursuit of renewable and degradable alternatives has emerged as a key research focus in materials science and industrial manufacturing [5]. Against this backdrop, bamboo fiber composites are gaining recognition as a promising replacement for conventional plastics, offering advantages such as renewability, lightweight properties, high specific strength, and low carbon emissions [6,7]. Bamboo fibers are widely available and biodegradable and can significantly reduce carbon footprints during usage, making them suitable for automotive manufacturing, construction materials, packaging, and other applications [8,9,10]. When integrated with a polypropylene matrix, bamboo fibers enhance the mechanical properties of the composite, meeting the automotive sector’s demands for lightweight and high-strength components [11,12,13]. The development and application of bamboo fiber-reinforced PP composites in automotive parts have become a focal point, drawing considerable interest from automakers globally. However, the choice of processing method is crucial to achieving optimal performance in bamboo fiber composites [14]. Although injection molding is prevalent in plastic processing, it presents challenges with bamboo fiber composites, including the thermal degradation of fibers, uneven distribution, and diminished mechanical performance. These limitations affect the practical application of bamboo fiber composites, highlighting the need to explore molding processes better suited to these materials.

Compared to injection molding, the compression molding process demonstrates notable technical advantages in the processing of plant fiber composites [15,16]. This method operates effectively at lower temperatures and pressures, significantly reducing the risk of the thermal degradation of fibers, thereby preserving the structural integrity and mechanical properties of the composite. Additionally, compression molding ensures an even distribution of fibers within the matrix, which not only helps maintain fiber length and strength but also enhances the isotropic mechanical characteristics of the material [17]. This process is particularly well suited for manufacturing large, uniformly thick components. Furthermore, compression molding offers high material utilization, generates minimal waste, and involves a relatively straightforward process with lower energy consumption, making it especially appropriate for medium- to low-volume production. Most importantly, compression molding retains the natural qualities of plant fibers, reducing degradation during molding and thereby increasing the material’s potential for sustainable and environmentally friendly applications.

While compression molding offers significant advantages over injection molding, it presents certain challenges [18,19,20]. The process is energy-intensive, requires complex parameter control, and faces difficulties in achieving the efficiency demands of large-scale production. Moreover, the nonlinear effects of molding parameters on material properties complicate process optimization for improved performance [21]. The carbon emissions from compression molding also remain insufficiently quantified, with studies highlighting the substantial impact of energy consumption and material selection on emissions [22,23,24]. For instance, higher temperatures and prolonged cycles significantly increase energy usage and carbon emissions [25]. Under carbon neutrality policies, optimizing process parameters and innovating techniques to simultaneously enhance composite performance and reduce carbon emissions are critical directions for further improvement [26].

Therefore, this study focuses on bamboo fiber-reinforced PP composites, addressing the current challenges of complex relationships between molding parameters and material performance, as well as the insufficient quantification of carbon emissions. A systematic solution is proposed using a three-factor, five-level orthogonal experimental design, where molding temperature, baking time, and holding time are selected as key variables, each set at five levels. Comprehensive experiments were conducted to analyze the nonlinear effects of different process parameters on composite performance. Furthermore, this study innovatively integrated a lifecycle carbon emission assessment, combining process optimization with environmental impact quantification for the first time. This study explored the contribution of various process parameters to carbon emissions and identified feasible emission reduction pathways. This research not only optimized the compression molding process for bamboo fiber-reinforced PP composites to enhance material performance but also provides a scientific basis for achieving low-carbon emissions in the molding process, addressing a critical research gap in the synergy of process parameter optimization and carbon emission control. Additionally, it offers technical support for the low-carbon and efficient application of plant fiber composites in the automotive industry, accelerating the green and sustainable development of automotive components. The findings also provide valuable insights into the development of high-performance, low-cost, and environmentally friendly materials, offering practical solutions for the automotive industry and beyond.

## 2. Materials and Methods

### 2.1. Materials

The bamboo fiber-reinforced PP composite used in this study was composed of bamboo fiber and PP. The polypropylene, model 5702P, was provided by SABIC (Saudi Basic Industries Corporation (Riyadh, Saudi Arabia)). Bamboo fibers were prepared in-house using the steam explosion method, with five-year-old Phyllostachys edulis (Moso bamboo) sourced from Yibin, Sichuan Province, China, as the raw material. Sodium hydroxide (NaOH), of analytical grade, was purchased from Chengdu Jinshan Chemical Reagent Co., Ltd. (Chengdu, China).

### 2.2. Sample Preparation

**Figure 1:**
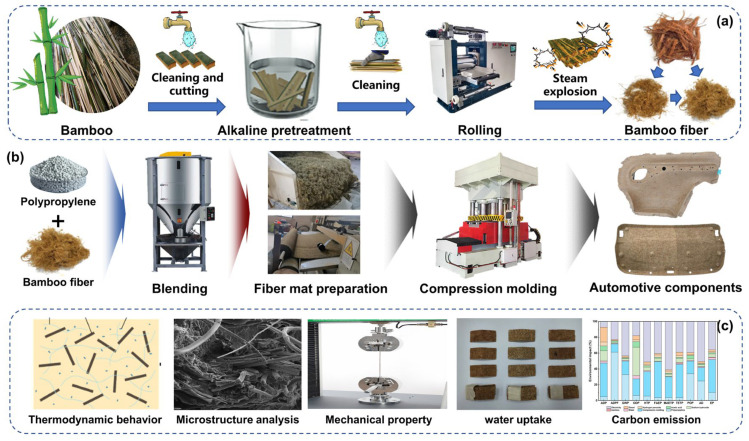
Compression molding process for plant fiber-reinforced PP composites. (**a**) Extraction process of bamboo fiber. (**b**) The compression molding process of bamboo fiber-reinforced polypropylene composite material. (**c**) Research content during the research process.

The compression molding process and experimental approach for the bamboo fiber-reinforced PP composite in this study are illustrated in Figure 1. Bamboo fibers were derived using the steam explosion method. Five-year-old Phyllostachys edulis bamboo was selected, cleaned, and sliced, and then pre-soaked in an alkaline NaOH solution to facilitate the separation of bamboo strands using a roller press, aiding in subsequent fiber extraction by steam explosion. The extracted bamboo fibers were screened and thoroughly mixed with polypropylene, followed by multiple processing steps, including opening, carding, needling, and trimming, to prepare bamboo fiber–polypropylene composite mats for compression molding. During the press molding process, the prepared composite felt is first preheated. Once it reaches the specified temperature, it is placed into the mold of the press molding machine for molding. After a certain period of pressure maintenance, the part is removed, and detailed trimming is performed to complete the manufacturing of the component. The molded components had a density of 1600 g/m^2^, a thickness of 2 mm, and a bamboo fiber-to-polypropylene mass ratio of 45% to 55%.

To address the process parameter challenges in the compression molding of bamboo fiber-reinforced PP composites, which show significant potential for development, a three-factor, five-level orthogonal experimental design was employed. Based on previous research and the press molding process, this study selected the preheating temperature of the plant fiber composite felt as Factor A, the preheating time of the plant fiber composite felt as Factor B, and the pressure maintenance time during the press molding of the plant fiber composite as Factor C. Each factor was set at five different levels, resulting in a total of 25 experimental combinations (L25 orthogonal array). Due to experimental resource constraints, 15 representative experimental combinations were selected from the 25 possible combinations in the L25 orthogonal array. This selection ensured the comprehensive exploration of factor-level interactions while optimizing resource utilization. Details of the selected combinations are shown in Table 1.

### 2.3. Characterization

#### 2.3.1. Characterization of Crystallization and Melting Behavior

A differential scanning calorimeter (DSC 214, Netzsch, Waldkraiburg, Germany) was employed to characterize the melting and crystallization behavior of the polymer blend system. The experimental procedure was as follows: precisely 10 mg of sample material was taken, sealed in an aluminum crucible, and placed in the DSC testing chamber under a high-purity nitrogen (N_2_) atmosphere for analysis. The starting temperature was set to 30 °C, increased to 220 °C at a rate of 10 K/min, and held for 5 min to eliminate the thermal history of the polymer. The temperature was then raised to 230 °C at the same rate, held for another 5 min, and subsequently cooled back to 30 °C at the same rate. The DSC curves and related data for the polymer were collected. The crystallinity (*χ_c_*) of PP was calculated using Equation (1):

(1) $$\chi_{c}=\frac{\Delta H_{m}}{\Delta H_{0}\times \omega }\times 100\%$$

where $\Delta H_{m}$ is the enthalpy of melting of PP (obtained from DSC testing); Δ*H*_0_ is the enthalpy of melting for fully crystalline PP (209 J/g); and *ω* is the mass fraction of PP.

#### 2.3.2. Characterization of Water Absorption Performance

Based on plastic water absorption measurement standard GB/T 1304-2008 (This standard is equivalent to the ISO international standard: ISO 62:2008) [27], bamboo fiber-reinforced PP composite samples were prepared to specified dimensions, with surfaces as flat and free of defects as possible. Each sample’s initial mass (*M*_1_) and initial thickness (*T*_1_) were measured using a precision balance (accuracy of 0.001 g) and a vernier caliper, ensuring the surface was free from contaminants and moisture. A water absorption test was then conducted by fully immersing the samples in distilled or deionized water at a controlled temperature of 23 °C ± 2 °C. The samples were soaked for periods of 2 h, 4 h, 6 h, 8 h, and 24 h, with enough water to ensure complete submersion without any part of the sample exposed above the water surface. After each specified soaking period, changes in the mass and thickness of the samples were measured. When removing the samples, excess water was gently blotted with absorbent paper without wiping, to prevent loss of adhered moisture. The mass change (*M*_2_) and thickness change (*T*_2_) of the soaked samples were then measured using the balance and vernier caliper.

The water absorption rate, *A*, can be expressed as

(2) $$A=\frac{M_{2}-M_{1}}{M_{1}}\times 100\%$$

The thickness swelling rate, *T*, can be expressed as

(3) $$T=\frac{T_{2}-T_{1}}{T_{1}}\times 100\%$$

#### 2.3.3. Characterization of Microstructure

A field emission scanning electron microscope (SEM JSM-IT800, manufactured by JEOL Ltd., Tokyo, Japan) was utilized to characterize the microstructure of the bamboo fiber-reinforced PP composite molded automotive parts. The specimens were subjected to a thorough cleaning process using high-pressure gas to eliminate surface contaminants. To preserve the integrity of the material interfaces’ original morphology, the samples were fractured in liquid nitrogen. Following the fracturing, the specimens were coated with a thin layer of platinum to enhance the electron scattering and improve the imaging quality in the SEM analysis. This coating process is crucial for obtaining clear images of the fracture surfaces and for distinguishing the features of the material’s microstructure. The preparation method allowed for an accurate observation of fiber orientation and distribution under varying environmental conditions. The microstructure analysis was further correlated with the mechanical properties to comprehensively evaluate the overall performance of the composites.

#### 2.3.4. Characterization of Mechanical Properties

The universal testing machine (CMT6104, manufactured by MTS Systems, Inc., San Jose, CA, USA) was employed to measure the tensile strength of standard tensile specimens according to the national standard GB/T 1040.1-2018 (This standard is equivalent to the ISO international standard: ISO 527-1:2019) [28] with a test rate of 20 mm/min. The flexural strength of standard flexural specimens was tested following the national standard GB/T 9341-2008 (This standard is equivalent to the ISO international standard: ISO 178:2019) [29], also at a rate of 20 mm/min. Each part underwent 10 tests for both tensile and flexural properties, with the results averaged across these tests. To evaluate impact strength, an Izod impact testing machine (XJUD-5.5, JJ-Test, Chengde, China) was used on standard V-notch impact specimens, based on the national standard GB/T 1843-2008 (This standard is equivalent to the ISO international standard: ISO 180:2023) [30], with an impact energy of 2.75 J. Each part was tested 10 times, and the results were averaged across these tests.

#### 2.3.5. Analysis of Life Cycle Assessment

**Figure 2:**
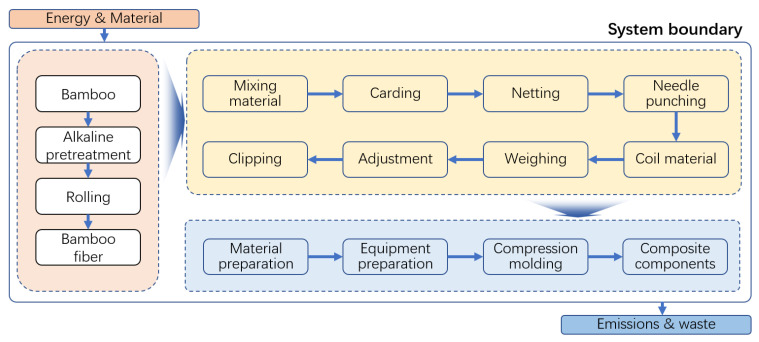
System boundaries of life cycle for compression molding of bamboo fiber-reinforced PP composites.

Using a life cycle assessment (LCA) approach, a cradle-to-gate study was conducted to evaluate the carbon emissions impact of bamboo fiber-reinforced PP composite compression-molded automotive parts. The system boundaries encompassed the entire life cycle, including bamboo fiber extraction, composite material preparation, and compression molding, as illustrated in Figure 2. This study employed the Centrum voor Milieukunde Leiden (CML) method to quantify and analyze the carbon emissions of the compression-molded bamboo fiber-reinforced PP automotive parts.

## 3. Results and Discussion

### 3.1. Influence of Process Parameters on Crystallization and Melting Behavior

**Figure 3:**
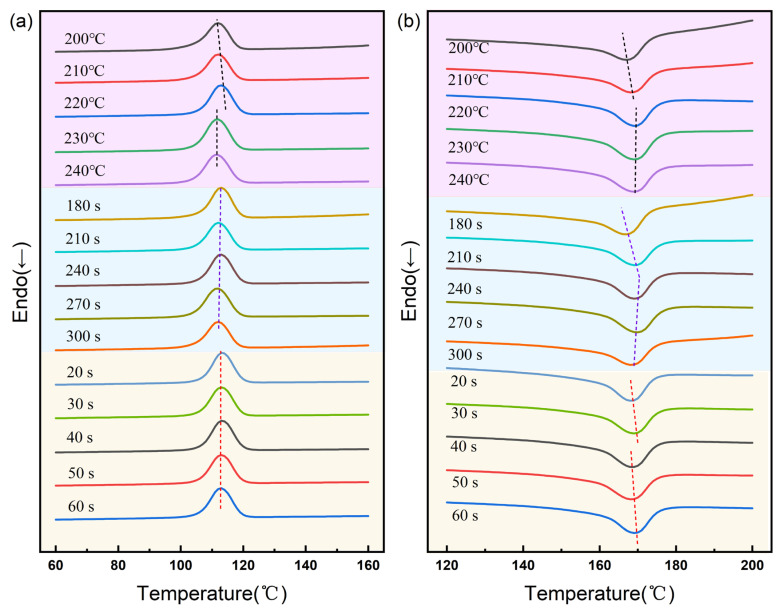
DSC curves of bamboo fiber-reinforced PP composite blend under different molding parameter conditions. (**a**) Crystallization curves. (**b**) Melting curves.

From the DSC curves of the blend system shown in Figure 3 and the thermal performance data of each composition presented in Table 2, it can be observed that adjustments to the preheating temperature have a slightly more significant impact on the melting point (*T_m_*) and crystallization temperature (*T_c_*) of bamboo fiber-reinforced PP composites, but a smaller impact on the degree of crystallinity (*X_c_*). At a preheating temperature of 200 °C, the *X_c_* value is relatively low, approximately 36.62%, leading to a slow crystallization rate and an inconspicuous induction effect on the bamboo fibers. However, as the temperature increases, the induction of crystallization on the fiber surface microstructure strengthens, and the polypropylene crystal structure becomes more stable, reaching an optimum at 220 °C. At this point, the value of $\Delta H_{m}$ also gradually increases with the temperature, resulting in higher crystallization and melting temperatures. Nevertheless, when the preheating temperature reaches 230 °C and above, excessive molecular chain activity may lead to unstable crystalline structures, with some crystals melting or defects forming, reducing the integrity of the crystal structure, and thus lowering the temperatures of the crystallization and melting peaks.

Preheating time has a relatively minor and irregular impact on the *T_c_* of bamboo fiber-reinforced PP composites, whereas its influence on the *T_m_* is more significant. It can be observed that as the preheating time increases, there is a certain elevation in the melting temperature. This can be interpreted as sufficient preheating time promoting the movement and alignment of molecular chains, which aids in forming more perfect crystal structures, thus requiring higher external temperatures for transition to the molten state. However, excessively long preheating times can lead to significant thermal history effects, resulting in unstable crystal structures and defects, which in turn lower the transition temperature to the molten state. Consequently, the data presented in the thermal performance table also show that during the process of increasing preheating time from 180 s to 300 s, the *X_c_* value of the composite material exhibits a peak-like variation. In contrast, the effect of holding time on the thermal properties of the composite material was not significantly observed in *T_m_* or *T_c_*. As the holding pressure time gradually increased, *X_c_* showed a trend of increasing initially and then decreasing, but the impact was minor.

### 3.2. Influence of Process Parameters on Overall Performance

During the compression molding process of plant fiber-reinforced PP composites, process parameters play a crucial role in influencing the overall performance of the final product. This section focuses on analyzing the performance variations in bamboo fiber-reinforced PP composites under different process parameters, specifically preheating temperature, preheating time, and holding time. Mechanical properties, including tensile strength, flexural strength, and impact strength, were assessed, along with microstructural characterization, to investigate the relationship between process parameters and product performance. Additionally, a water absorption test was conducted, considering the specific characteristics of plant fiber composites, to determine the optimal combination of process parameters for enhancing the material’s overall mechanical performance. The experimental results provide a scientific basis for optimizing the compression molding process and reveal the critical role of each process parameter in improving the composite’s comprehensive performance.

**Figure 4:**
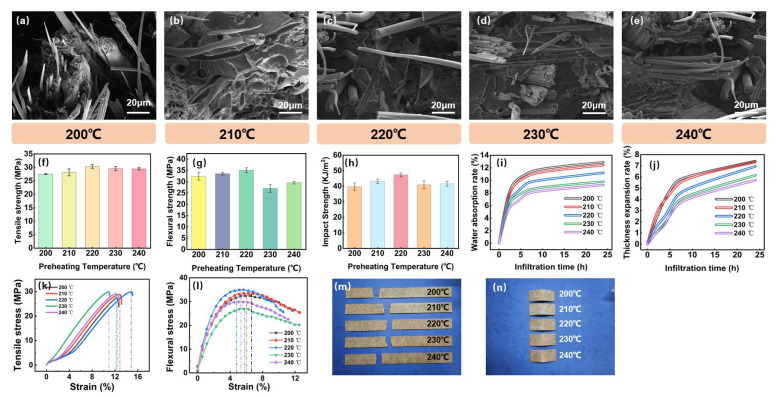
Effects of preheating temperature on the properties of the composite. (**a**–**e**) Microstructural changes in the composite at different preheating temperature, (**f**) tensile strength, (**g**) flexural strength, (**h**) impact strength, (**i**) water absorption rate, (**j**) thickness expansion rate, (**k**) tensile stress, (**l**) flexural stress, (**m**) tensile specimen, and (**n**) flexural specimen.

The preheating temperature of composite materials is crucial to the quality of their processing performance. In this study, we designed five sets of experimental schemes at preheating temperatures of 200 °C, 210 °C, 220 °C, 230 °C, and 240 °C to explore the impact of preheating temperature on the material’s molding performance. The results are shown in Figure 4. Specifically, Figure 4f illustrates that as the preheating temperature increases from 200 °C to 240 °C, the tensile strength of the material first rises and then slightly decreases. Corresponding to the stress–strain curves in Figure 4l, it is observed that appropriately increasing the preheating temperature promotes the enhancement of the material’s mechanical properties. Specifically, at 200 °C, the tensile strength of the material is relatively low, with only 27.51 MPa. However, when the temperature is increased to 220 °C, the tensile strength can be improved by 10.32%, which is a significant enhancement in strength. Additionally, by examining the bonding between bamboo fibers and polypropylene in Figure 4a–e, it was found that within this temperature range, the interfacial bonding between the molecular chains and bamboo fibers is improved, leading to an increase in the overall structural stability of the material. However, when the temperature is further increased to 230 °C and 240 °C, there is a slight decrease in tensile strength. This may be due to the excessively high temperature causing a deterioration in the interfacial bonding between the bamboo fibers and the matrix, or some damage to the internal molecular chain structure of the material. This phenomenon is similar to that observed by Xie et al. in the thermoforming process of carbon fiber-reinforced polymer composites [31], highlighting the importance of preheating temperature for subsequent molding processes.

From Figure 4g, it can be observed that the preheating temperature has a similar effect on the flexural strength of the material. Further analysis in conjunction with the flexural stress–strain curves in Figure 4m reveals that at 200 °C, the flexural strength of the material is relatively low, indicating that at low temperatures, the internal structure of the material has not yet fully developed, as shown by the micromorphology in Figure 4a–e. However, when the temperature is increased to 220 °C, the flexural strength reaches a maximum value of 35.12 MPa, indicating that the molecular chains and bamboo fibers of the material have been effectively aligned and closely bonded at this temperature. However, when the temperature is further increased to 230 °C and 240 °C, the flexural strength decreases by 15–20%. This is associated with the degradation of bamboo fibers at excessively high temperatures. The degradation of bamboo fibers at higher temperatures leads to a decrease in performance, a phenomenon also observed in the thermoforming process of glass fiber-reinforced polymer composites [32]. However, further analysis still reveals that at 240 °C, the flexural performance of the composite material shows a certain improvement. This phenomenon confirms that at excessively high temperatures, bamboo fibers may undergo carbonization under certain conditions, thus leading to a temporary increase in the mechanical properties of the composite material. However, continuous heating and carbonization should not be considered a means to enhance material performance [33]. Therefore, 220 °C remains an ideal preheating temperature to ensure that the material has excellent flexural strength. We provide the morphologies of the test specimens at different temperature parameters in Figure 4k,n. Moreover, as depicted in Figure 4h, the variation in preheating temperature has a more significant impact on the impact strength of the material. At 220 °C, the impact strength reaches its peak. At this temperature, the molecular chains are fully extended, and the interfacial bonding and stress transfer efficiency between the bamboo fibers and the polypropylene matrix are optimized.

The effects of preheating temperature on the water absorption properties of the product are shown in Figure 4i,j. The test results of water absorption rate and thickness expansion rate indicate that the preheating temperature significantly influences the water absorption properties of bamboo fiber-reinforced PP composites. At lower preheating temperatures (200 °C and 210 °C), the materials exhibit higher water absorption and expansion rates. In conjunction with the microstructures shown in Figure 4 and previous research on the water absorption of plant fiber composites [34], this suggests the impact of different temperature parameters on the porosity of the composite materials. Conversely, at higher preheating temperatures (230 °C and 240 °C), there is a significant reduction in water absorption, and the expansion rates are at their lowest, with the internal structure being more compact and the dimensional stability at its best. Therefore, to enhance the moisture resistance and dimensional stability of bamboo fiber-reinforced composites, it is recommended to prefer a preheating temperature range of 230 °C to 240 °C during the thermoforming process. This temperature range is optimal for achieving the desired properties in the final product.

**Figure 5:**
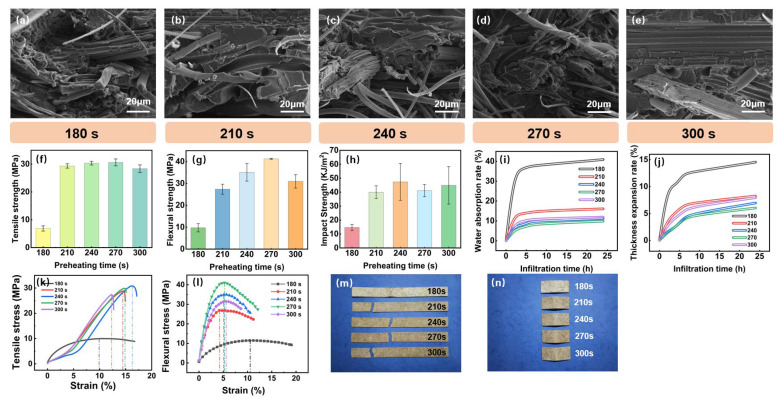
Effects of preheating time on the properties of the composite. (**a**–**e**) Microstructural changes in the composite at different preheating time, (**f**) tensile strength, (**g**) flexural strength, (**h**) impact strength, (**i**) water absorption rate, (**j**) thickness expansion rate, (**k**) tensile stress, (**l**) flexural stress, (**m**) tensile specimen, and (**n**) flexural specimen.

The preheating time for composite materials, like the preheating temperature, is crucial and should be used in conjunction [35]. The effects of preheating time on the material are depicted in Figure 5. Upon integrating the mechanical property test results under microscopic morphology and conducting a comprehensive evaluation of the material’s stress–strain curves, it is observed that a preheating time of 180 s results in a sharp decline in performance. At this point, the tensile strength is 6.87 MPa, which is approximately 22% of the performance under other preheating temperature settings. The structural characteristics of the tensile specimens after testing, as shown in Figure 5m,n, reveal that under the 180 s condition, the specimens are loosely structured, barely formable, and exhibit extremely poor mechanical properties. This underscores the significance of sufficient preheating time for the material’s mechanical performance.

Further analysis indicates that the comprehensive mechanical properties of the material are most notably enhanced when the preheating time is within the range of 210 to 270 s. Within this interval, as demonstrated in Figure 5f–h, the tensile, flexural, and impact strengths of the material reach their optimum, suggesting that the molecular chains and bamboo fibers achieve the best alignment and bonding state. However, when the preheating time exceeds 270 s, the performance tends to stabilize or slightly decrease, possibly due to the accumulation of internal stress leading to a weakening of structural stability. Therefore, it is recommended that the preheating time be controlled between 210 and 270 s during the thermoforming process to achieve the best material performance. This optimal preheating duration ensures that the material’s molecular chains and bamboo fibers are properly aligned and bonded, resulting in the highest mechanical performance.

The test results of the water absorption rate and thickness expansion rate in Figure 5i,j demonstrate that preheating time significantly affects the water absorption properties of bamboo fiber-reinforced PP composites. At a shorter preheating time (180 s), the material exhibits higher water absorption and expansion, indicative of poor interfacial bonding between the fibers and the matrix. In conjunction with the previous mechanical property analysis, it is evident that insufficient preheating time directly leads to inadequate fusion between the material matrix, resulting in decreased mechanical performance and issues such as increased porosity [36]. Conversely, when the preheating time is extended to 270 to 300 s, there is approximately a 15% reduction in water absorption and about a 5% decrease in expansion rate. At this duration, the internal structure of the material is more compact, offering optimal dimensional stability. Therefore, to enhance the material’s resistance to water and improve dimensional stability, it is recommended to select a preheating time range of 270 to 300 s during the thermoforming process. This preheating duration is optimal for achieving a material with superior water resistance and dimensional stability, as it allows for better interfacial bonding and a denser internal structure.

**Figure 6:**
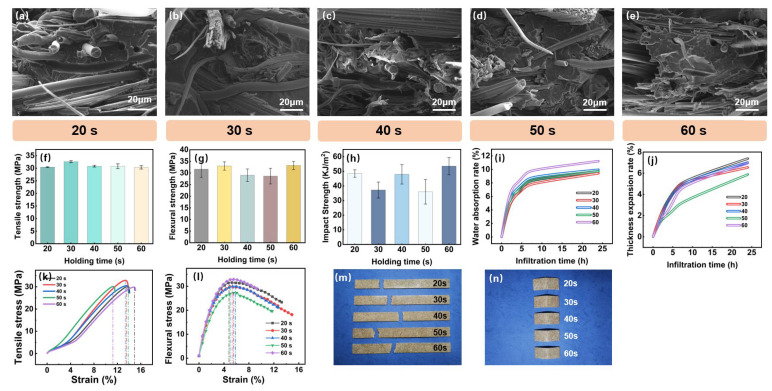
Effects of holding time on the properties of the composite. (**a**–**e**) Microstructural changes in the composite at different holding times, (**f**) tensile strength, (**g**) flexural strength, (**h**) impact strength, (**i**) water absorption rate, (**j**) thickness expansion rate, (**k**) tensile stress, (**l**) flexural stress, (**m**) tensile specimen, and (**n**) flexural specimen.

The impact of different holding times on the performance of bamboo fiber-reinforced polypropylene composites during the thermoforming process is illustrated in Figure 6. It can be observed that as the holding time increases, the overall change in the material’s tensile and flexural properties is not significant, and a trend similar to that of the initial preheating temperature and preheating time is noted, with a rise followed by a decrease. However, the effect on impact strength is more significant, with distinct variations in impact performance under different holding time parameters. Thus, the influence of holding time is notably less than that of the previously set parameters, which aligns with findings from previous studies on the molding of long fiber-reinforced composites [37].

Furthermore, a detailed analysis in conjunction with the stress–strain curves in Figure 6k,l reveals that an appropriate holding time can enhance the material’s elastic modulus and flexural modulus, thereby achieving a more desirable mechanical performance. In summary, a holding time of 30 s is considered reasonable, as it allows for favorable mechanical and water absorption properties. Therefore, it is recommended that the holding time be controlled at 30 s during the thermoforming process to achieve optimal material performance. This controlled holding time ensures that the material exhibits good mechanical properties and water resistance, making it suitable for various applications where these characteristics are crucial.

In summary, through a systematic analysis of the tensile, flexural, and impact properties and the water absorption of bamboo fiber-reinforced polypropylene composites under various preheating temperatures, preheating times, and holding times, the optimal combination of process parameters can be identified as follows: a preheating temperature of 220 °C, a preheating time between 240 and 270 s, and a holding time of 30 s. At a preheating temperature of 220 °C, the molecular chains and bamboo fibers are most optimally aligned within the matrix, significantly enhancing the material’s mechanical properties while reducing water absorption and thickness expansion rates, demonstrating the best moisture resistance and dimensional stability. A preheating time within the range of 240 to 270 s contributes to the rearrangement of the material’s internal microstructure and the strengthening of interfacial bonding, leading to peak tensile, flexural, and impact strengths, while maintaining low water absorption and expansion rates, indicating the best compactness and stability of its structure. When the holding time is set to 30 s, the material exhibits the highest comprehensive performance, effectively releasing internal stress and forming a uniform microstructure, which improves overall mechanical properties and moisture resistance.

### 3.3. Impact Analysis of Life Cycle Assessment

**Figure 7:**
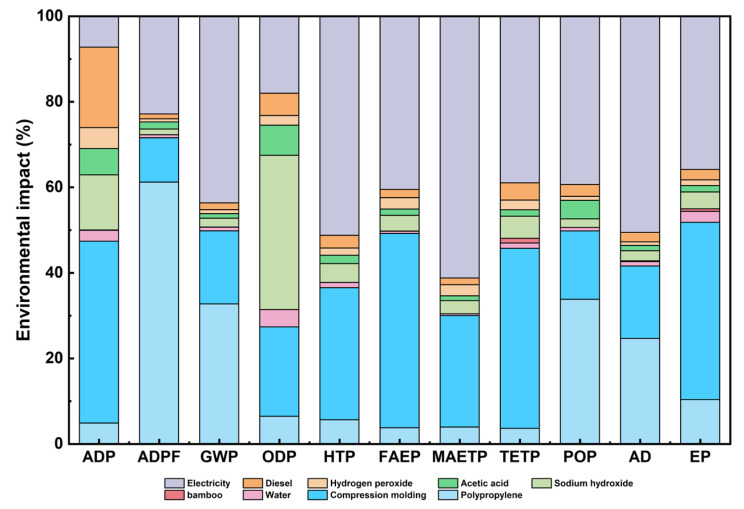
Life cycle assessment analysis of bamboo fiber-reinforced PP composite material compression molding.

Based on the CML evaluation standard, a detailed analysis of 11 environmental impact indicators was conducted for the life cycle of bamboo fiber-reinforced polypropylene composites during the compression molding process. These indicators include Abiotic Depletion (ADP), Abiotic Depletion (fossil fuels) (ADPF), Global Warming (GWP), Ozone Layer Depletion (ODP), Human Toxicity (HTP), Freshwater Aquatic Ecotoxicity (FAEP), Marine Aquatic Ecotoxicity (MAETP), Terrestrial Ecotoxicity (TETP), Photochemical Oxidation (POP), Acidification (AD), and Eutrophication (EP). The system boundaries covered the entire cradle-to-gate life cycle, including plant fiber extraction, composite preparation, and compression molding, assessing resource consumption and environmental load. The results are shown in Figure 7. Overall, polypropylene production and the compression molding process are the primary sources of environmental burden throughout the life cycle, significantly impacting several key environmental indicators.

Firstly, from the perspective of resource consumption, polypropylene production is the primary contributor to abiotic resource depletion, accounting for 42.5%. In contrast, the natural environmental benefits of bamboo fiber are highlighted, underscoring the green and sustainable potential of developing bamboo fiber-reinforced PP composites. Additionally, energy consumption during the compression molding process constitutes 10.35% of total resource consumption, emphasizing the importance of optimizing energy use and improving energy efficiency in future molding processes.

In today’s global carbon neutrality context, examining greenhouse gas emissions is increasingly essential. Under the Global Warming Potential (GWP) indicator, electricity consumption is the largest source of greenhouse gas emissions, accounting for 43.65%. This is followed by polypropylene production at 32.75%, and the compression molding process at 17.08%. This indicates that greenhouse gas emissions throughout the life cycle are highly dependent on the efficiency of electricity usage. Enhancing energy efficiency in the compression molding and polypropylene production stages, especially through the integration of clean energy sources, could significantly reduce carbon emissions.

Regarding Human Toxicity Potential (HTP) and Freshwater Aquatic Ecotoxicity Potential (FAEP), electricity consumption accounts for 51.2% of HTP, while the impact of the compression molding process on freshwater ecotoxicity is 45.42%. This highlights the profound environmental and health impacts of pollutant emissions resulting from energy consumption and material use in manufacturing processes. Therefore, in future process design and optimization, in addition to material substitution, attention should be paid to the type and cleanliness of energy sources to mitigate toxicity impacts.

For Ozone Depletion Potential (ODP), the compression molding process contributes approximately 20.92%, while polypropylene production impacts Acidification Potential (AD) by 24.68%. These indicators are highly sensitive to process parameters, such as temperature and time, suggesting that the fine control and optimization of molding parameters, such as through appropriately reducing temperature and shortening molding time, can lead to substantial environmental benefits.

## 4. Conclusions

This study conducted an in-depth investigation and optimization of the compression molding process for bamboo fiber-reinforced PP composites used in automotive interior and exterior components. The aim was to explore the factors affecting the molding performance of environmentally friendly automotive parts, identify the optimal molding parameters, and assess their impact on material properties and life cycle carbon emissions. Through a three-factor, five-level experimental design, we systematically analyzed the effects of preheating temperature, preheating time, and holding pressure time on the thermal properties, mechanical properties, water absorption, and microstructure of the material. Additionally, we innovatively introduced research on resource and environmental impacts within the life cycle. This study found that the crystallinity of bamboo fiber-reinforced PP composites was not significantly affected by the three factors, but their melting and crystallization temperatures were influenced to some extent. Among the three factors, preheating time and temperature had a greater impact on the comprehensive properties of the material, while the effect of holding pressure time was relatively minor. Optimal mechanical and water absorption properties were achieved when the preheating temperature was around 220 °C, the preheating time was 270 s, and the holding pressure time was approximately 30 s, with a more complete integration of fibers and matrix in the microstructure. Furthermore, life cycle assessment revealed that the current compression molding process, energy consumption, and polypropylene materials are the main contributors to environmental issues. Future efforts can focus on optimizing the compression molding process, using clean energy, and promoting the use of plant fiber-reinforced polymer composites to advance sustainable development in the automotive parts industry. This also provides significant technical support for green manufacturing and sustainable development in automotive parts and other fields, accelerating the realization of lower carbon and more efficient molding processes and offering new solutions for achieving global carbon neutrality goals.

## Figures and Tables

**Table 1:** Orthogonal experimental table for compression molding parameters of plant fiber-reinforced polypropylene.

Experimental Group	Preheating Temperature (°C)	Preheating Time (s)	Molding Pressure (MPa)	Holding Time (s)
1	200	240	20	60
2	210		20	
3	220		20	
4	230		20	
5	240		20	
6	220	180	20	60
7		210	20	
8		240	20	
9		270	20	
10		300	20	
11	220	240	20	20
12			20	30
13			20	40
14			20	50
15			20	60

**Table 2:** Thermal performance data of each composition.

Composition	$T_{m}$ (°C)	$T_{c}$ (°C)	${\Delta H}_{m}$ (J/g)	$X_{c}$
200 °C	167.0 ± 0.1	111.9 ± 0.2	38.27 ± 0.1	36.62 ± 0.1
210 °C	168.4 ± 0.1	112.0 ± 0.2	38.47 ± 0.1	36.81 ± 0.1
220 °C	169.2 ± 0.1	112.8 ± 0.2	38.96 ± 0.1	37.28 ± 0.1
230 °C	169.1 ± 0.1	111.7 ± 0.2	38.75 ± 0.1	37.08 ± 0.1
240 °C	169.1 ± 0.1	111.5 ± 0.2	38.73 ± 0.1	37.06 ± 0.1
180 s	166.8 ± 0.1	112.9 ± 0.2	38.19 ± 0.1	36.55 ± 0.1
210 s	169.3 ± 0.1	112.0 ± 0.2	38.64 ± 0.1	36.98 ± 0.1
240 s	169.2 ± 0.1	112.8 ± 0.2	39.03 ± 0.1	37.35 ± 0.1
270 s	169.9 ± 0.1	111.7 ± 0.2	39.12 ± 0.1	37.44 ± 0.1
300 s	168.4 ± 0.1	112.0 ± 0.2	38.56 ± 0.1	36.90 ± 0.1
20 s	168.3 ± 0.1	113.3 ± 0.2	38.48 ± 0.1	36.82 ± 0.1
30 s	168.9 ± 0.1	113.0 ± 0.2	39.09 ± 0.1	37.41 ± 0.1
40 s	168.6 ± 0.1	113.3 ± 0.2	39.1 ± 0.1	37.42 ± 0.1
50 s	168.4 ± 0.1	113.0 ± 0.2	38.88 ± 0.1	37.21 ± 0.1
60 s	169.2 ± 0.1	112.8 ± 0.2	38.84 ± 0.1	37.17 ± 0.1

## Data Availability

Due to the requirements of enterprise cooperation and experimental data sharing, the data in this article can be provided as requested.
